# Effects of Extreme Precipitation to the Distribution of Infectious Diseases in Taiwan, 1994–2008

**DOI:** 10.1371/journal.pone.0034651

**Published:** 2012-06-21

**Authors:** Mu-Jean Chen, Chuan-Yao Lin, Yi-Ting Wu, Pei-Chih Wu, Shih-Chun Lung, Huey-Jen Su

**Affiliations:** 1 Department of Environmental and Occupational Health, Medical College, National Cheng Kung University, Tainan, Taiwan; 2 Research Center for Environmental Changes, Academia Sinica, Taipei, Taiwan; 3 Department of Occupational Safety, Foundation of Taiwan Industry Service, Taipei, Taiwan; 4 Department of Occupational Safety and Health, Chang Jung Christian University, Tainan, Taiwan; University of Oxford, Viet Nam

## Abstract

The incidence of extreme precipitation has increased with the exacerbation of worldwide climate disruption. We hypothesize an association between precipitation and the distribution patterns that would affect the endemic burden of 8 infectious diseases in Taiwan, including water- and vector-borne infectious diseases. A database integrating daily precipitation and temperature, along with the infectious disease case registry for all 352 townships in the main island of Taiwan was analysed for the period from 1994 to 2008. Four precipitation levels, <130 mm, 130–200 mm, 200–350 mm and >350 mm, were categorized to represent quantitative differences, and their associations with each specific disease was investigated using the Generalized Additive Mixed Model and afterwards mapped on to the Geographical Information System. Daily precipitation levels were significantly correlated with all 8 mandatory-notified infectious diseases in Taiwan. For water-borne infections, extreme torrential precipitation (>350 mm/day) was found to result in the highest relative risk for bacillary dysentery and enterovirus infections when compared to ordinary rain (<130 mm/day). Yet, for vector-borne diseases, the relative risk of dengue fever and Japanese encephalitis increased with greater precipitation only up to 350 mm. Differential lag effects following precipitation were statistically associated with increased risk for contracting individual infectious diseases. This study’s findings can help health resource sector management better allocate medical resources and be better prepared to deal with infectious disease outbreaks following future extreme precipitation events.

## Introduction

Global climate disruption appears to have increased the intensity of tropical cyclones, including typhoons in Southeast Asia, hurricanes in Central America and the Southeast United States, consequently resulting in much greater probability of extreme precipitation [1]–[5]. Observational and projection studies also suggest that turbulent atmospheric activity has and will intensify the regional precipitation in the past, present and future [4]–[6].

Extreme precipitation events, increasing the amount of regional precipitation and flooding, have heightened the risk and concern over the transmission of various infectious diseases which may affect their distribution and chances of becoming epidemics [6]–[12]. During periods of heavy precipitation, local water quality can be seriously compromised via diverse means, a significant one being the cross-contamination of water sources due to infiltration and inflow between sewage and water pipes [11]. Contaminated water sources for drinking and recreation, due mostly to flooding after extreme precipitation, have been associated with water-borne diseases outbreaks and epidemics [7]–[8], [10]–[16]. Extreme precipitation also can leave pools of stagnant water that create optimal breeding grounds and growth environments for vectors and hosts, including mosquitoes, mites, rodents and insects [9]–[10], [12].

Significant associations between increased precipitation and greater incidences of contracting water-borne infectious diseases were found in various geographic regions [13], [15]–[25]. When data on 548 water-borne disease epidemics and outbreaks reported by the U.S. Environmental Protection Agency (EPA) between 1948 and 1994 were analysed; a statistical relationship was identified between precipitation and water-borne disease, in which 51% of waterborne disease outbreaks were linked to precipitation levels above the 90^th^ percentile, and 68% to levels above the 80^th^ percentile [16]. In Canada, precipitation was implicated with water-borne disease outbreaks [15], [20]. Precipitation of greater than the 93^rd^ percentile increases the risk of water-borne disease outbreaks by a factor of 2.283 [15]. Cholera and leptospirosis epidemics, in Bangladesh and India, were reported to be associated with heavy precipitation [25]. Outbreaks of gastrointestinal infections usually result when people are exposed to increased amounts and diverse types of pathogens, including *Giardia lamblia*, *Cryptosporidium* [21], and enterovirus [23]–[24] after heavy precipitation. Furthermore, previous studies indicated the impact of precipitation on mosquito-borne, tick-borne, and rodent-borne diseases such as dengue fever, West Nile fever, Japanese encephalitis, Chikungunya fever, malaria, *Lyme borreliosis*, and hantavirus infection, especially when there was the concurrent presence of high temperatures [19], [26]–[29].

As global climate change is predicted to increase the trend of extreme precipitation events occurring is more likely, so determining the role of weather in the incidence of infectious disease is a public health priority [13]. A better understanding of the relationship between disease and extreme precipitation can be an important first step toward finding ways to mitigate the risk of diseases and the diseases’ impact on communities [11]. However, only a limited number of studies have explored the relationship between daily precipitation and climate-related infectious diseases, performed in such places as the United Kingdom [22] and North America [11], [13]–[16]. Most of these observations were based on case reports and some localized databases, so only examined the relationship in specific areas [11], [13], [16], [22]. In this study, a database, using the Geographic Information System (GIS) as the analysis platform, integrated nationwide Taiwanese weather and health surveillance data. Poisson regression using a generalized additive mixed model was applied to examine the relationships between daily extreme precipitation and the number of reported cases for 8 climate-related infectious diseases, including water-borne infectious diseases (hepatitis A, enteroviruses, bacillary dysentery, leptospirosis and melioidosis), and vector-borne infectious diseases (scrub typhus, dengue fever, Japanese Encephalitis).

## Results

The Pearson product-moment correlation identified that extreme precipitation events were associated with the occurrence of 8 infectious diseases with lags of 0–70 days. Table 1 provides descriptive statistics for the registered cases of 8 infectious diseases, including time periods, disease categories, incubation periods, case numbers, and specifically significant lag days following precipitation.

**Table 1:** Characteristics of climate-related infectious diseases in Taiwan, 1994–2008.

	Water-borneDiseases					Vector-borne Diseases		
	Hepatitis A	Enteroviruses^a^	Bacillary dysentery	Leptospirosis	Melioidosis	Scrub typhus	Dengue fever	Japanese encephalitis
**ICD-9**	701	749	004	100	025	812	061	620
**Period**	1994–2008	1994–2008	1994–2008	2006–2008	2007–2008	1994–2008	1994–2008	1994–2008
**Incubation**	15–50 days	02–10 days	1 week	2–30 days	2 days+	06–21 days	3–14 days	5–15 days
**No. of cases**								
Total	3358	1970	4828	116	44	3110	11178	314
<130 mm	3347 (99.67%)	1940 (98.48%)	4745 (98.28%)	110 (94.83%)	42 (95.45%)	3074 (98.84%)	11072 (99.05%)	306 (97.45%)
130–200 mm	0009 (00.27%)	0021 (01.07%)	0015 (00.31%)	002 (01.72%)	01 (02.27%)	017 (00.55%)	00062 (00.55%)	003 (00.96%)
201–350 mm	0001 (00.03%)	0007 (00.36%)	0037 (00.77%)	004 (03.45%)	01 (02.27%)	016 (00.51%)	00044 (00.39%)	005 (01.59%)
>350 mm	0001 (00.03%)	0002 (00.10%)	0021 (00.43%)	000 (00.00%)	00 (00.00%)	0003 (00.10%)	00000 (00.00%)	000 (00.00%)
**Lag day**	lag_28_	lag_07_	lag_07_	lag_14_	lag_14_	lag_21_	lag_70_	lag_14_

a With severe complications, as adopted by the Taiwan Center for Disease Control.

Adjusted relative risks for the extreme precipitation categories, as they relate to the baseline control precipitation category (<130 mm), are presented in Table 2. Heavy precipitation (130–200 mm) was a significant risk factor for enterovirus infections (RR = 2.45, 1.59–3.78) and dengue fever (RR = 1.96, 1.53–2.52). The results further indicated that torrential rain events (201–350 mm) were a critical determinant for the reporting of most infectious diseases except for Hepatitis A and enterovirus infections. Extreme torrential rain (>350 mm) impacted enterovirus infections (RR = 5.981, 1.474–23.760) and bacillary dysentery (RR = 7.703, 5.008–11.849). The trend tests demonstrated noteworthy associations between precipitation levels and the reporting of enterovirus infection and Japanese encephalitis (P_trend_<0.001), and even stronger linear relationships between precipitation and bacillary dysentery, dengue fever, and leptospirosis (P_trend_<0.0001).

**Table 2:** Relative risks (RRs) and 95% CIs for 8 infectious diseases in Taiwan, 1994–2008^a^.

	Regular <130 mm	Heavy 131–200 mm	Torrential 201–350 mm	Extreme torrential >350 mm	P_trend_
**Hepatitis A** (lag_28_)	1.000	1.135 (0.589, 02.187)	0.208 (0.029, 01.481)	1.533 (0.216, 10.904)	0.1697
**Enteroviruses** b (lag _7_)	1.000	2.452** (1.592, 03.777)	1.429 (0.679, 03.006)	5.981* (1.474, 23.760)	0.0002
**Bacillary dysentery**(lag _7_)	1.000	0.751 (0.452, 01.248)	2.851** (2.060, 03.946)	7.703** (5.008, 11.849)	<0.0001
**Leptospirosis** (lag_14_)	1.000	2.958 (0.720, 12.143)	8.541** (3.083, 23.685)	N.A.	<0.0001
**Melioidosis** (lag_14_)	1.000	10.010* (1.303, 78.899)	10.010* (1.303, 78.899)	N.A.	0.1062
**Scrub typhus** (lag_21_)	1.000	1.233 (0.765, 01.989)	1.782* (1.089, 02.915)	1.842 (0.593, 05.722)	0.0011
**Dengue** (lag_70_)	1.000	1.962** (1.527, 02.522)	2.094** (1.555, 02.819)	N.A.	<0.0001
**Jap. encephalitis** (lag_14_)	1.000	1.546 (0.494, 04.834)	4.258* (1.749, 10.363)	N.A.	0.0038

Note. CI  =  confidence interval. N.A.: non-available.

* *p*<0.05.

** *p*<0.001.

a The multivariable mixed generalized additive model was adjusted for parameters of calendar month and location (352 townships), which was adjusted for loss function of the multiple-lag effects of temperature.

b With severe complications adopted by Taiwan CDC.

**Figure 1:**
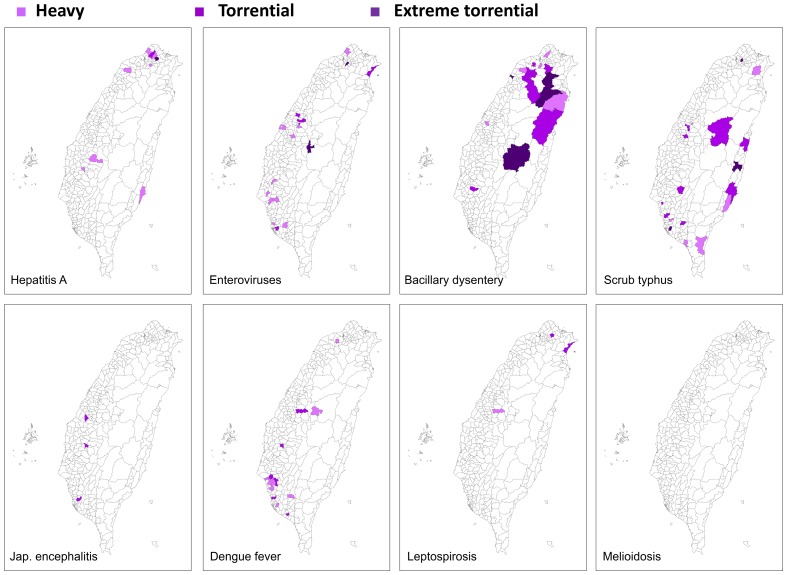
Risk maps of the 8 climate-related infectious diseases following extreme precipitation events were generated when the analysis was integrated with the GIS system. The townships that had a significant association between the outbreak of the specified disease with the category of extreme precipitation events are marked on each map. Townships that had significant associations with heavy precipitation are marked in light purple, with torrential precipitation are marked in purple, while those that only had significant associations with extreme torrential precipitation are marked in dark purple.

**Figure 2:**
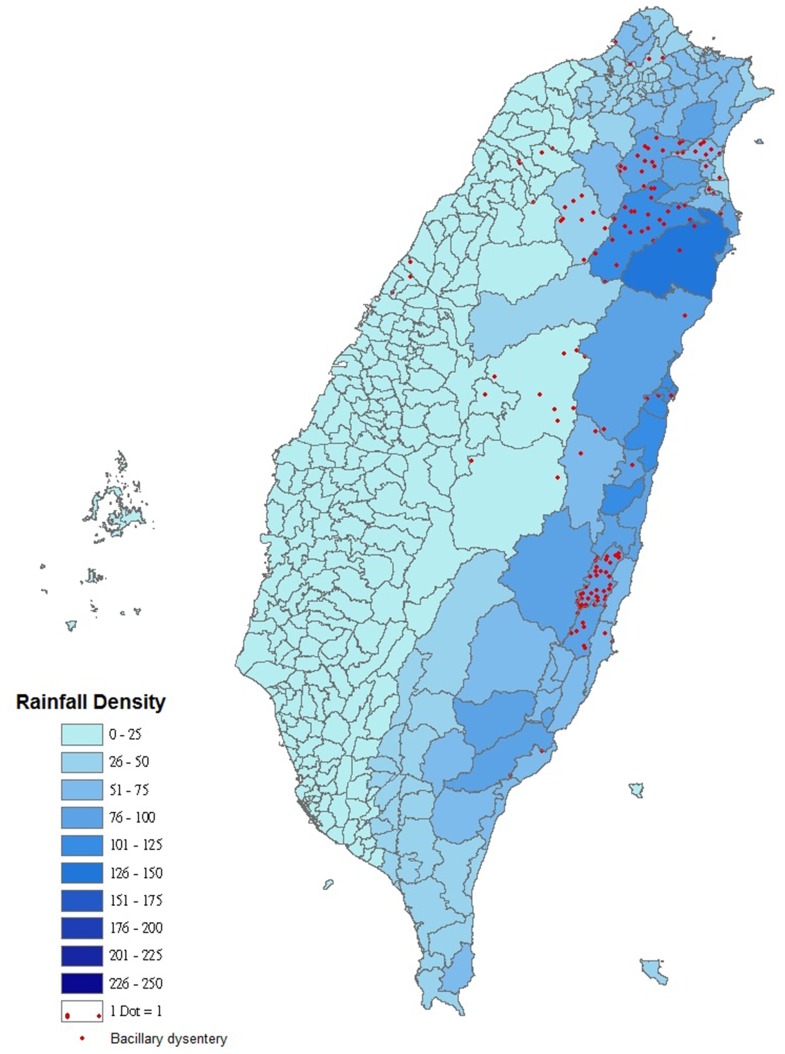
Distribution of daily mean precipitation (in mm after Typhoon Nari) and the individual cases of bacillary dysentery with a 7-day lag after Typhoon Nari, 2001.

A relative risk measure for each disease was created individually for each township. This allowed for determination of the specific townships at risk for particular precipitation-disease outbreaks. Figure 1 shows the vulnerable townships that had precipitation related disease outbreaks, categorized based on precipitation and the 8 climate-related infectious diseases. Figure 2 is a specific case situation highlighting the intensity of precipitation and bacillary dysentery with a 7-day lag after Typhoon Nari, 2001.

From the 12 socioeconomic, demographic and geographical characteristics examined, the proportion of natives were significantly higher (+15.5%; P_t-test_ = 0.0042) along with the average elevation of a township (+339 m; P_t-test_<0.0001) in those townships that had a significant relationship between extreme precipitation and the occurrence of Bacillary dysentery as compared to townships that did not have this particular significant relationship. In addition, the relative number of elderly living alone was higher (+27.4%; P_t-test_ = 0.0182) in those townships at risk for Scrub Typhus following extreme precipitation events. Furthermore, townships with higher population densities were the ones associated with extreme precipitation related enterovirus infections (+94888.0; P_t-test_ = 0.0037) and dengue fever (+59253.6; P_t-test_ = 0.0212).

## Discussion

Our study is the first to demonstrate the quantitative risk of the relationship between extreme precipitation events during the last 15 years and 8 climate-related infectious diseases at the national level of a subtropical country. A statistically significant association was found between different forms of extreme precipitation and diseases, including enterovirus infection, Japan encephalitis, scrub typhus, dengue fever, and leptospirosis in Taiwan. The maps further display at the regional level the specific townships (of the 352 total townships in mainland Taiwan) that are impacted by extreme precipitation-mediated outbreaks of each infectious disease.

For intestinal infectious diseases, the positive relationships between precipitation and enterovirus infection and bacillary dysentery were similar to previous reports [7], [23]–[24], [30]–[32]. Enteroviruses were more likely to be flushed from unsaturated zones and contaminate groundwater after a short spell of heavy rain [23]. Studies in China found that precipitation may affect the transmission and incidence of food-borne diseases like bacillary dysentery [30], [32]. The results indicated that precipitation may influence pathogens, contamination of drinking water, and then influencing the diseases epidemic [30].

For vector-borne diseases, higher precipitation has been associated with dengue fever, scrub typhus, and Japanese encephalitis [26], [29], [33]. An important study indicated that mosquito population dynamics, and so subsequent disease transmission, would be susceptible to precipitation frequency [34]. When the frequency of precipitation event occurrences match the natural frequency of the mosquito reproductive cycle, then quick phase locking and resonance can result allowing mosquito populations to grow most efficiently and exponentially [34]. However, our study suggested that precipitation >350 mm/day probably destroyed the vector habitats, and there may be an upper threshold value for the positive effect of precipitation on disease vectors. It can be speculated that the emphasis on controlling vector-borne infectious diseases can probably be shifted away from it and to focusing on the prevention of outbreaks of water-borne infections after extreme torrential precipitation events.

Although precipitation events usually last on the order of days, in preceding studies, the analytic lags of precipitation effects were mostly performed on the weekly or monthly scale [13], [28], [35]. Even though studies have demonstrated a daily lag, nevertheless, they were often for short-term periods or limited locations [11], [13], [36]. In our study which the data covers a 15 year period, the specific lag effects between showed significant correlations between daily precipitation and registered case dates. The eruption of 7 infectious diseases appeared within 7–28 days, while dengue fever seemed to appear 70 days after an extreme precipitation event (The 70 day lag effect we observed for dengue is not unexpected, since the imago of *Aedes Aegypti* (the main vector of dengue fever in Taiwan) is about 9 days, its lifespan is about 30 days, and the incubation period is 3 to 14 days).

**Figure 3:**
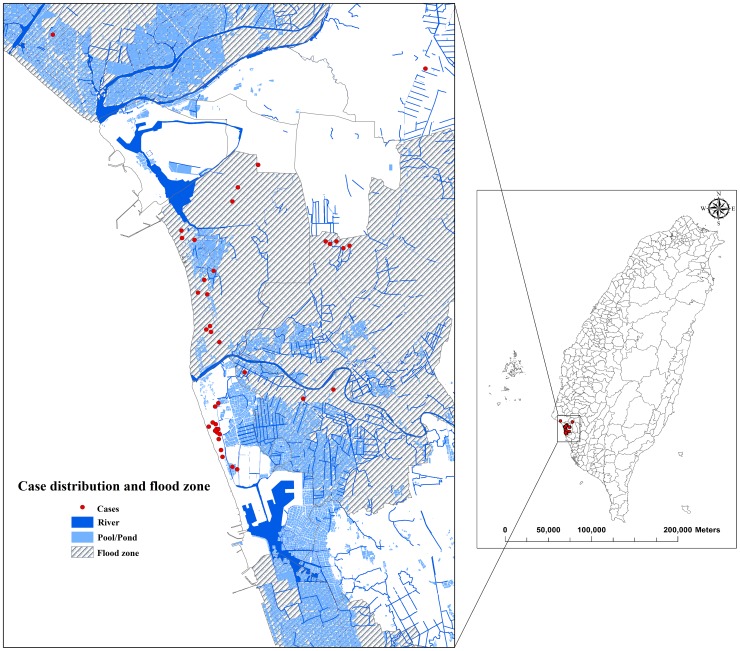
Case distribution of melioidosis after Typhoon Haitang, 2005. There were 40 cases in southern Taiwan after Typhoon Haitang. 97.5% cases resided in the flooded areas and 70% of cases were in proximate contact of mud or flooding waters.

In Australia, the United States, Germany, India, Malaysia, and Thailand, it has been reported that high-intensity precipitation is associated with reports of leptospirosis or melioidosis [36]–[44]. Our results could not support a distinct quantity of risk for leptospirosis and melioidosis because mandated reporting did not begin until 2005. But, several Taiwanese publications reported a melioidosis outbreak approximately 2 weeks after typhoon Haitang (maximum precipitation was over 1000 mm/day) in 2005 [45]–[46]. A field survey found that 97.5% of cases were reported in flooded areas (Figure 3). Super typhoon Morakot brought extreme precipitation in 2009; the accumulated precipitation in southern Taiwan in the typhoon Morakot period was nearly 3000 mm over 3 days [47]–[48]. Leptospirosis and melioidosis outbreaks in flood zones have also been reported after extreme precipitation [49]–[50]. Consequently, we presume that higher precipitation has relationships with occurrences of higher risk for leptospirosis and melioidosis outbreaks.

The results provide quantitative risk values (Table 2) and identify the geographic distributions (Figure 1) of 8 infectious diseases as preliminary evidence linking precipitation with those diseases occurrence. However, the occurrence or spread of a disease was also affected by other factors such as public health services, population density and demographics, land use changes, and socioeconomic conditions. This study tried to explore the basic relationships between socioeconomic factors in precipitation-related vulnerable townships. The proportion of native Taiwanese, elevation and proportion of elderly living alone were indicators of those regions vulnerable to disease outbreaks due to precipitation related events. It is important to link climatic impact to these other factors so adequate medical plans, health promotion policies and prevention campaigns can be properly implemented to prevent or at least minimize disease outbreaks in the future.

From 1961 to 2005 the incidence of intense precipitation (defined as the top 10% precipitation events) had doubled [51]. A previous study also provided independent evidence in support for significant increases in the number and/or size of strong global tropical cyclones [52]. This means extreme precipitation and water-related catastrophes such as floods, mudflows and landslides may become more frequent in the future. [2], [6], [51]–[52]. Extreme weather conditions are a global phenomenon making extreme precipitation an important issue not only in Taiwan but also in many other regions worldwide.

In general, for regions vulnerable to extreme precipitation related disease outbreaks regional risk assessments should be performed, as has been done for Taiwan in this study, in order to determine what local areas are at greatest risk, and identify those specific associated characteristics that contribute to the outbreak. This analysis provides decision makers with essential information to guide their proper implementation of technology- and knowledge-based strategies to best manage precipitation related infectious disease outbreaks. Technology-based approaches include creating better, more efficient monitoring and prediction systems, improving emergency medical aid, promoting environmental conservation, and improving emergency alert systems [19]. Knowledge-based approaches include determining the most appropriate distribution of medical resources, and educating the public to increase awareness of hygiene and health and how to protect oneself as this pertains to epidemic control under extreme weather conditions [28].

The infrequent occurrence of the most extreme torrential precipitation in Taiwan limited the statistical power of the analysis in this study; it is possible that more significant associations between extreme precipitation and the occurrence of the diseases we studied exist. This study did not specifically identify the particular vulnerable clusters, for each infectious disease, present within the entire general population. The population characteristics addressed by the t-test comparisons suggest what particular characteristics define each specific cluster. In our future studies, using a cohort follow-up design, the particular vulnerable clusters for infectious disease in a region will be identified for one of the specific infectious diseases examined in this study. This information would be helpful to medical agencies that want better targeted interventions [53]. Future studies will also identify the mechanisms, such as drinking water contamination, responsible for extreme precipitation associations with infectious disease outbreaks.

Previous studies have demonstrated that extreme precipitation has an impact on the occurrence of infectious diseases. The model presented here successfully demonstrated relationships between extreme levels of precipitation and the incidences of water-borne and vector-borne infectious diseases. This is the first study to use nationwide records to conduct spatial statistical analysis focusing on high-spatial-resolution. Our study exhibits the first systematic and quantitative risk assessment of the relationship between daily extreme precipitation and 8 infectious diseases at national and regional levels. There were significant correlations between specific infectious diseases and the occurrences of extreme precipitation events in Taiwan. Results also highlight the local characteristics that may exacerbate infectious disease outbreaks following extreme precipitation conditions. This study’s findings provide critical information that aid in the creation of effective health policies, and the establishment of relevant technology- and knowledge-based strategies so Taiwan is better prepared for the climatic emergencies of the future.

## Methods

### Island-wide Meteorological Data

Hourly data from 33 fixed–site monitoring stations, and approximately 300 auto-monitoring stations from the CWB (Taiwan’s Central Weather Bureau), that had complete precipitation and temperature records from 1994 to 2008, were obtained. Since the weather monitoring stations in Taiwan are mostly clustered in urban areas along the east coastline, we could not obtain direct measures of precipitation distribution throughout the entire island, especially from rural and mountain areas. In order to generate representative daily precipitation and temperature measures for each of the 352 total townships in Taiwan, we used the Ordinary Kriging geo-statistical method as part of the ArcGIS 9.3 system. This method is useful to generate optimal spatial-linear temperature and precipitation data, by interpolating the temperature and precipitation at an unobserved location using the actual values from nearby locations. We extrapolated precipitation and temperature data in a stepwise procedure at a resolution of 1×1 square kilometres since this was the smallest geographical unit for disease notification [54]–[56]. This approach allowed us to obtain the best-possible representative precipitation and temperature for each township.

### Definition of Extreme Precipitation

As defined by the Taiwan CWB, precipitation events were categorized as follows: regular: daily accumulated precipitation below 130 mm; heavy rain: at 130–200 mm; torrential rain: at 201–350 mm; and extreme torrential rain: above 351 mm [57]. Based on these definitions, the probability of extreme precipitation occurring was much less than 1% since the averaged 99^th^ percentile of precipitation for all 352 townships in Taiwan over the 15 year period was 92.80 mm/day (Lower quartile: 0.04 mm/day, upper quartile: 4.63 mm/day). The heavy rain events in Taiwan were 17.4 days, torrential rain events were 11.7 days and extreme torrential rain events were 1.8 days of each township happened in the 15 years.

### Case Definition of Infectious Disease

The computerized database, with recorded daily registry of the occurrence of the 8 mandatory-notified infectious diseases, along with age, gender, township of residence, and the time of disease onset for each case, was retrieved from the Taiwan CDC (Center Disease Control) for the period from 1994–2008, with a signed agreement for confidentiality; all diagnoses were confirmed by the reference laboratory at the Research and Diagnostic Center of the Taiwan CDC. Only the “indigenous” cases for each disease, after verification of travel history, disease onset, and virus subtype classification by the National Virus Diagnosis Laboratory in Taiwan, were analysed [58].

### Demographic and Socioeconomic Factors

Twelve selected socioeconomic and demographic characteristics for each township, including medical resources (the number of clinics and doctors) and demographic characteristics (percentage of native Taiwanese, elderly, elderly living alone, and disabled persons), type of employment (either service; industry; agricultural; other), and socioeconomic conditions (household ownership, uneducated population, unemployment rate, and percentage of labourers working outside their county of residence) were estimated using the health statistics obtained from the Department of Health, as determined from the national census taken in 2000 (performed once per decade) [54].

### Statistical Analysis

The database included the aggregated daily case registry of 8 infectious diseases and weather monitoring data of precipitation and temperature from 1994 to 2008 for each township. Pearson product-moment correlation analyses assessed the specific lag effects (at weekly intervals) between the number of case occurrences and the direct measures of precipitation amount and temperature levels. Only weekly lag intervals were considered, since these are easier to interpret and act upon from a disease management standpoint. Determination of the length of lag time designated for analysis took into account the incubation period of the pathogen by only considering lag times in excess of the minimum incubation period such as the time needed for vector-associated infection to occur and biologically mature. The number of daily registered cases was a dependent variable (count data), and the daily accumulated precipitation level was the major independent variable. Poisson regression, using a generalized additive model (GAM), has been the suggested method of analysis in time-series studies [59]. GAM allows a Poisson regression to be fit as a sum of nonparametric smooth functions of predictor variables. The purpose of GAM is to maximize the predictive quality of a dependent variable, “Y,” from various distributions by estimating archetypical functions of the predictor variables that are connected to the dependent variable.

Poisson regression, using a Generalized Additive Mixed Model (GAMM), was used to evaluate the multiple-lag effects of stratified precipitation levels on specific diseases [59]. Furthermore, all models were adjusted for the multiple-lag effects of daily temperature, month, and township for evaluating the associations between categorized extreme precipitation and diseases, with further trend tests performed to examine linear associations between levels of precipitation and outbreaks of each disease.

The functions are as follows:

Overall statistical function
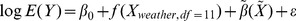



Function 1: analysis averaged over all of Taiwan
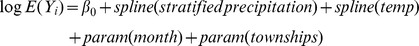



Function 2: analysis performed on each township
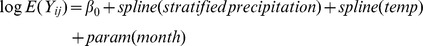
where 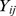
is the vector of daily case counts for the *i*  = 1, 2,…, 8 infectious diseases and *j*  = 1, 2,…, 352 townships of Taiwan. 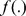 represented the smoothing functions, which have to satisfy standardized conditions such as 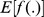  = 0. 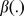 were parameters that included categorized precipitation amounts, month, and townships, which showed the multiple-level effects of precipitation, monthly patterns and geographical location [59]–[60]. Function 2 was performed individually on our data for each township. We mapped those townships that had significant associations between extreme precipitation events and disease outbreaks to the GIS system and formulated a risk map. In addition, t-tests were used to contrast the differences in basic socioeconomic characteristics in the precipitation-related vulnerable townships to the townships that did not show a significant relationship between precipitation and disease outbreaks.
